# O-RADS MRI risk stratification system: pearls and pitfalls

**DOI:** 10.1186/s13244-023-01577-5

**Published:** 2024-02-14

**Authors:** Stephanie Nougaret, Leo Razakamanantsoa, Elizabeth A. Sadowski, Erica B. Stein, Yulia Lakhman, Nicole M. Hindman, Aurelie Jalaguier-Coudray, Andrea G. Rockall, Isabelle Thomassin-Naggara

**Affiliations:** 1 Department of Radiology, Montpellier Cancer Institute, Montpellier, France; 2 Montpellier Research Cancer Institute, PINKcc Lab, U1194 Montpellier, France; 3 Sorbonne Université, INSERM UMR S 938 (CRSA - 75012), Assistance Publique des Hôpitaux de Paris, Hopital Tenon, Service IRIS, Paris, France; 4 Departments of Radiology, Obstetrics and Gynecology, University of Wisconsin School of Medicine and Public Health, 600 Highland Ave, E3/372, Madison, WI 53792-3252 USA; 5 Department of Radiology, University of Michigan Health System, 1500 E. Medical Center Drive UH B1 D502, Ann Arbor, MI 48109-5030 USA; 6 Departments of Radiology, Memorial Sloan Kettering Cancer Center, New York, USA; 7 New York University School of Medicine, 660 First Avenue, New York, NY 10016 USA; 8 Departments of Radiology, Institut Paoli Calmettes and CRCM, Aix Marseille Université, , 13009 Marseille, France; 9 Division of Surgery and Cancer, Imperial College London, Hammersmith Campus, London, UK

**Keywords:** O-RADS, MRI, Ovarian lesion, Stratification

## Abstract

In 2021, the American College of Radiology (ACR) Ovarian-Adnexal Reporting and Data System (O-RADS) MRI Committee developed a risk stratification system and lexicon for assessing adnexal lesions using MRI. Like the BI-RADS classification, O-RADS MRI provides a standardized language for communication between radiologists and clinicians. It is essential for radiologists to be familiar with the O-RADS algorithmic approach to avoid misclassifications. Training, like that offered by International Ovarian Tumor Analysis (IOTA), is essential to ensure accurate and consistent application of the O-RADS MRI system. Tools such as the O-RADS MRI calculator aim to ensure an algorithmic approach. This review highlights the key teaching points, pearls, and pitfalls when using the O-RADS MRI risk stratification system.

**Critical relevance statement** This article highlights the pearls and pitfalls of using the O-RADS MRI scoring system in clinical practice.

**Key points**

• Solid tissue is described as displaying post- contrast enhancement.

• Endosalpingeal folds, fimbriated end of the tube, smooth wall, or septa are not solid tissue.

• Low-risk TIC has no shoulder or plateau. An intermediate-risk TIC has a shoulder and plateau, though the shoulder is less steep compared to outer myometrium.

**Graphical Abstract:**

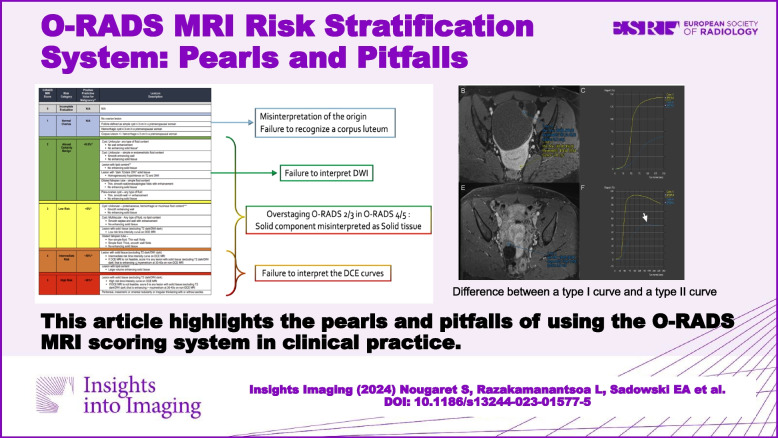

## Introduction

In 2021, the American College of Radiology (ACR) Ovarian-Adnexal Reporting and Data Systems (O-RADS) MRI Committee created and introduced an evidence-based lexicon and risk stratification system for assessing adnexal lesions using MRI [1]. The O-RADS MRI scoring system evaluates the adnexal lesion’s MRI appearance and provides a numerical risk score from 1 to 5, with 0 indicating incomplete or uninterpretable imaging. In this system, the higher the risk score, the greater the risk of malignancy.

A clear benefit of the O-RADS MRI classification system, similar to the universally accepted ACR Breast Imaging Reporting and Data System classification is the adoption of a uniform and descriptive lexicon, which can facilitate communication between radiologists and referring physicians [2]. This was recently confirmed by a metaanalysis including 4520 adnexal lesions showing that O-RADS MRI had a sensitivity and specificity over 90% in characterizing adnexal lesions [3]. However, in a recent EURAD study, Thomassin-Naggara et al. highlighted several common errors in O-RADS MRI assessment and analyzed the reason for the misclassified cases [4]. Out of a total of 1502 lesions, 139 (9.2%) were misclassified. The primary reasons for misclassification were misinterpretation of solid tissue (*n* = 104) and errors in identifying the origin of the lesion (*n* = 35) [4]. The authors emphasized the need for radiologist training in O-RADS MRI, similar to the training offered by the International Ovarian Tumor Analysis (IOTA) group [5]. In addition, tools such as the recently implemented O-RADS MRI calculator (https://oradsmricalc.com) will help radiologists to adhere to the essential algorithmic approach. In this review, we highlight the pearls and pitfalls of using the O-RADS MRI scoring system in clinical practice.

## Brief overview of O-RADS MRI

The ACR O-RADS MRI Committee has recently introduced an MRI lexicon and risk stratification system to assess adnexal lesions. This initiative aims to standardize image acquisition and reporting, ensuring a consistent reporting lexicon and improved agreement in image interpretation [1]. Notably, this lexicon resembles the ACR O-RADS ultrasound (US) lexicon and risk stratification system introduced by Andreotti et al. [6]. Developed through a modified Delphi process, the ACR O-RADS MRI lexicon comprises seven categories of descriptors agreed upon by imaging experts through consensus.[1]. The major lexicon categories include lesion shape or contour, signal intensity, type (cyst without or with a solid component, solid), fluid (simple, non-simple), solid component (solid tissue or non-solid tissue), and extra-ovarian findings [1].

## Pearls and pitfalls

- Technical aspects

### Acquisition pearls

Scheduling the exam according to menstrual cycle is not necessary. The main goal of patient preparation is to reduce motion artifacts and should include fasting (at least 3 h prior to the examination) and the use of an antiperistaltic agent (20 mg butylscopolamine or 1 mg of glucagon if not contraindicated).

**Fig. 1:**
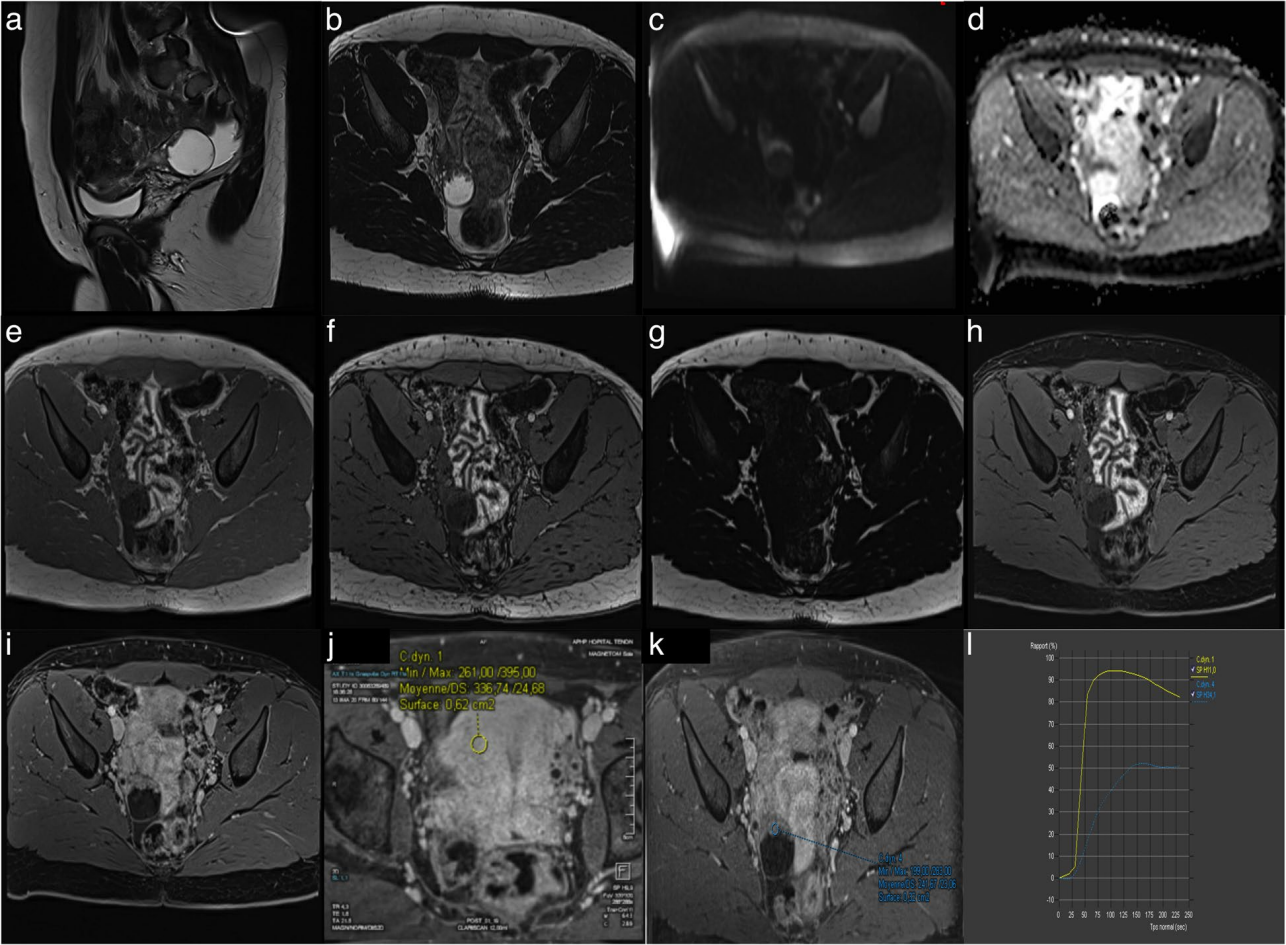
Optimal MRI protocol to perform O-RADS MRI Risk scoring. **a** Sagittal T2-weighted sequence without fat saturation. **b** Axial T2-weighted lombo-pelvic sequence. **c** Axial DWI lombo-pelvic scan (*b*: 1400 s/mm^2^). **d** Corresponding axial ADC map. **e** Axial unenhanced T1-weighted in-phase. **f** Axial unenhanced T1-weighted opposed-phase. **g** Axial unenhanced T1-weighted Fat sequence. **h** Axial unenhanced T1-weighted water sequence. **i** Axial late-gadolinium T1-weighted sequence. **j**, **k** DCE T1-weighted sequence with myometrial curve (yellow curve) and region of interest within the solid ovarian tissue (blue curve) (**l**). This was classifified as an O-RADS MRI 4, and hypothesized to represent a serous borderline cystadenoma

Phased Array body coil should be used for image acquisition. The patient is imaged in the supine position and the following imaging sequences should be acquired though the pelvis for optimal adnexal lesion characterization (Fig. 1, Table 1):

- Sagittal T2-weighted without fat saturation (slice thickness, ≤ 4 mm)
- Axial T2-weighted without fat saturation (section thickness, ≤3 mm)
- Axial unenhanced T1-weighted in-, opposed-phased, fat and water sequence (section thickness, ≤ 4 mm)
- Axial abdo-pelvic DWI scan (section thickness 4–5 mm, low *b*-values: 0 or 50 − high *b*-value: 1000–1200)
- Three-dimensional non-dynamic T1-weighted gadolinium sequence with fat sat (slice thickness 3 mm) without contrast
- Three-dimensional dynamic contrast-enhanced (DCE) MRI T1-weighted sequence (if any enhancing solid tissue within the adnexal lesion): 3D axial (15 cm–15 s–3 mm)
  - Maximal slice thickness of 3 mm
  - Axial plane, in line with the T2-weighted sequence and DWI scan for ease of cross correlation of the signal characteristics
  - Minimum temporal resolution of 15 s per acquisition
  - Subtraction allows the suppression of any pre-contrast high T1-weighted signal intensity, whether the DCE is acquired with or without fat saturation.
  - The acquisition needs to start prior to contrast injection (10 s prior to contrast injection) and should continue for at least 3 min.
- If DCE MRI is not feasible, an alternative option is to perform a T1WI series at 30–40 s post-contrast injection. It is important to note that this approach will have limitations in assessing adnexal lesions with enhancing solid tissue, which can only be categorized as O-RADS MRI 4 or 5.

**Table 1:** Summary, pearls and pitfalls of imaging protocol

**Sequence**	**Aim**	**Pitfall (solution)**
Sagittal T2-weighted without fat saturation	Tissue characterization Multiplanar analysis Pelvic anatomy analysis Location of the mass analysis Shape, size and component of the mass analysis	1. Intravenous contrast injection (if very-dark signal on T2-W imaging, perform DWI scan, to search for both low T2, DWI scan signal to avoid gadolinium injection) 2. Failure to recognize intermediate T2W signal (combine with DWI to evaluate the need for contrast injection analysis) 3. Failure to identify the anatomical origin of the pelvic mass (look for “specific” anatomic sign, landmarks displacement and ipsilateral ovary visualization) 4. Failure to identify small tissular component as papillary projections (use thin slices < 4 mm)
Axial T2-weighted lombo-pelvic sequence	Same as above Extra-pelvic organ analysis (kidney, liver, vascular structure, lymph node)	1. Failure to identify extra-pelvic disease (analyze all the slices of the sequence)
Axial unenhanced T1-weighted in-phase sequence	Tissue characterization	1. Wrong analysis of the component if hypersignal T1-W (analyze complementary T1W sequence to determine blood, high-protein or fat content)
Axial unenhanced T1-weighted water sequence	Tissue characterization	1. Wrong analysis of the fat component (analyze the loss of signal and correlate with T1-weighted fat sequence if doubt) 2. Wrong identification of mucinous component (correlate with morphological sequence to look for loci and high-signal T2W) 3. Wrong identification of pus component (correlate with inflammatory clinical findings, perform gadolinium sequence and DWI scan) 4. Wrong identification of colloid component (correlate with morphological sequence to look for loci and low-signal T2W) 5. Wrong analysis between endometriotic and other hemorrhagic content (correlate with clinical findings, T2W sequence “shading” and T1W “rim”)
Axial abdominal and -pelvic DWI scan	Tissue characterization Identification of pathological lymph node	1. Technical issue with the acquisition (perform the sequence with a minimum upper *b*-value of 1000 s/mm^2^ , section thickness < 4 mm and check the dark DWI signal of the bladder with high-*b*-value before interpretation) 2. Analyze the apparent diffusion coefficient to conclude (do not use ADC value to interpret adnexal masses) 3. Analyze the dark T2/DWI partially (analyze the whole tumor to avoid missing high-signal component) 4. Misclassifying the normal ovary with ovarian tumor (correlate to morphological T2W sequence)
Three-dimensional non-dynamic T1-weighted gadolinium sequence	Tissue characterization (identify tissular component)	1. Technical issue with the acquisition (ensure perform pre-contrast acquisition to enable subtraction sequences and use section thickness < 3 mm) 2. Failure to identify Rokitansky nodule or thin and regular septations (correlate with morphological T2W and T1W sequence) 3. Failure to identify endosalpingial folds and papillary projections in the context of inflammatory pelvic disease (correlate with clinical findings, morphological T2W sequence and DWI scan) 4. Failure to identify a physiological fimbria end of the tube (correlate with multiplanar T2W and search for stellar morphology) 5. Failure to identify hair, calcifications, debris (use T1W in-, opposed-, fat, and water sequence) 6. Failure to identify normal ovarian parenchyma (use T2W and DWI scan)
Three-dimensional DCE T1-weighted sequence	Tissue characterization using tissular component and enhancement analysis	1. Technical issue with the acquisition (perform the sequence with a spatial resolution of 3 mm and temporal resolution of 15 s, place the reference ROI on the outer myometrium avoiding arcuate vessels. Start contrast injection 10 s prior and last for at least 3 min) 2. Technical issue with the curve drawing (analysis must be performed using percentage of enhancement of relative enhancement not absolute) 3. Technical issue with the curve drawing in mixt tumor (perform subtraction sequence to put the ROI) 4. Failure to identify a shoulder and a plateau to differentiate low and intermediate-risk TIC (do not consider the slope of the curve but search for a shoulder and a plateau) 5. Being inconclusive in the context of hysterectomy (analyze the 30–40 s post-contrast sequence to search for an early enhancement of the mass) 6. Drawing enhancement curve in the context of adnexal torsion (correlate with clinical findings and T2W morphological sequences and DWI scan) 7. Systematically drawing enhancement curve in the context of dermoid cyst and struma ovarii (intrinsic component of the tumor can lead to misdiagnosis of a malignant tumor)

Table 1 summarizes the O-RADS MRI protocol, the added value of each sequence, and pitfalls of acquisition.

b. Interpretation

**Fig. 2:**
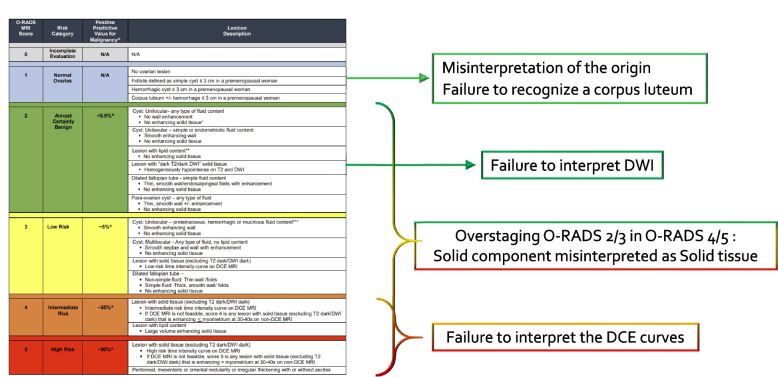
O-RADS classification with most common errors. Please refer to reference 7 for positive predictive values

There are six risk score categories in the O-RADS MRI risk stratification system which are detailed below and in Fig. 2.

#### O-RADS MRI score 0

**Table:** 

**Definition:** Lesions that have undergone incomplete or inadequate MRI evaluation are included in this category.	

This category includes lesions that are partly imaged or imaged without intravenous contrast because the assessment of contrast enhancement is critical for risk stratification. It also includes situation where the recommended protocol was not followed.

#### O-RADS MRI score 1

**Table:** 

**Definition**: Normal ovaries or ovaries with physiologic observations, such as a follicle, corpus luteum cyst, and hemorrhagic cyst measuring ≤ 3 cm in premenopausal women.	

### Pitfall 1

This category emphasizes the key role that MRI plays in identifying the origin of the pelvic lesion. In the EURAD study, among 1194 patients referred for MRI, 64 (5.4%) patients did not have a pelvic lesion, and 130 (8.6%) had a non-adnexal lesion [4, 7]. Furthermore, twelve adnexal lesions were misclassified as non-adnexal, whereas three were classified as adnexal in origin but were in actuality non-adnexal lesion. Size contributed greatly to the misclassification as ten (83.3%) of the former 12 misclassified adnexal lesions were larger than 5 cm [4, 7]. These results illustrate the potential pitfall of this category, i.e., misdiagnosis of non-adnexal lesion versus adnexal lesion.

**Fig. 3:**
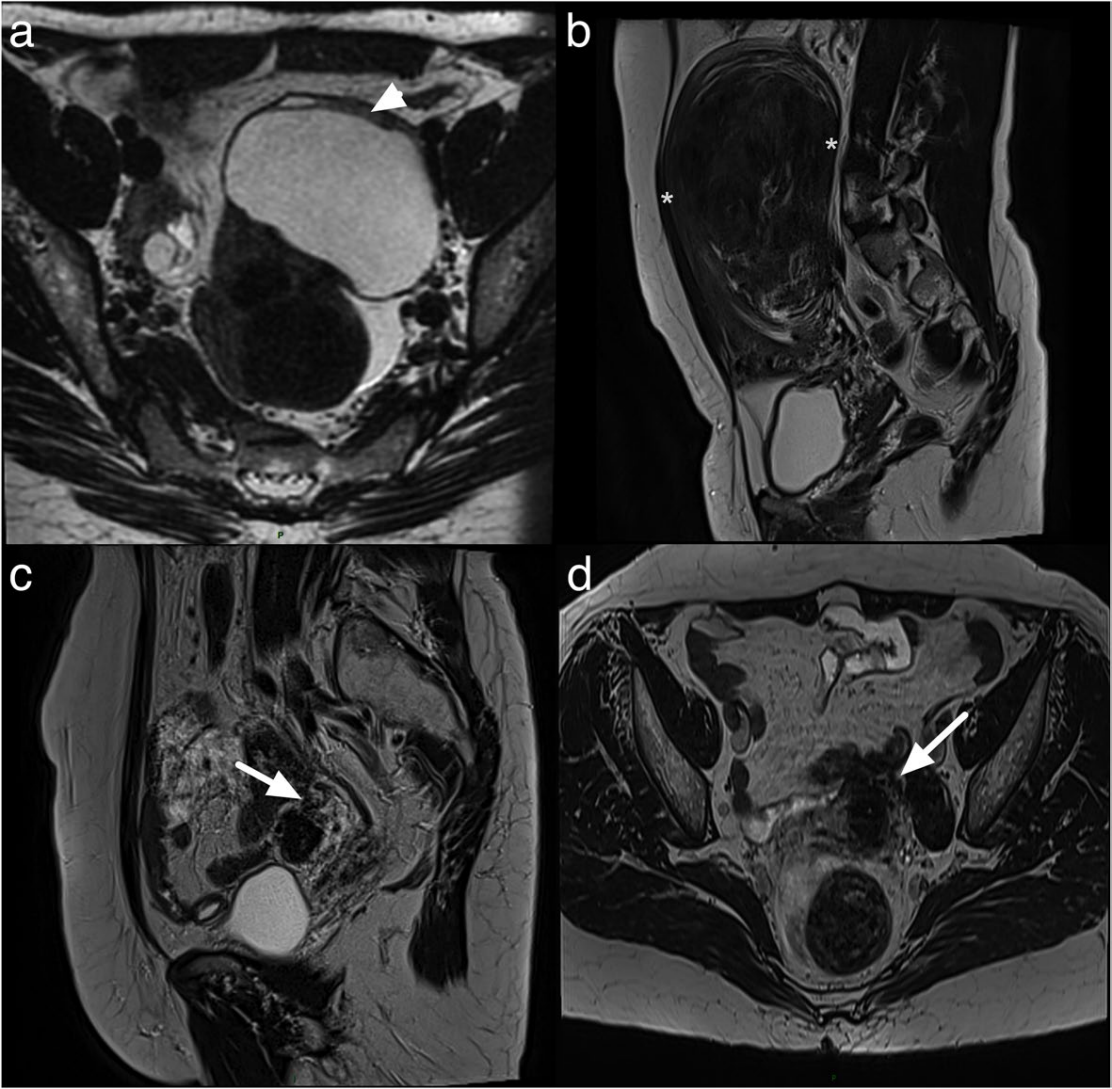
Useful sign to evaluate the relationship between a lesion and adjacent organ: **a** Axial T2-weighted sequence with a “beak sign” (white arrow head). **b** Sagittal T2-weighted sequence showing a “claw sign” (white star). **c**, **d** Sagittal and Axial T2-weighted sequence with “bridging vessel sign” (arrow)

Pearls to correctly assess the origin of the lesion:

1. Identifying normal adnexa on the same side as the lesion can rule out adnexal origin. Diffusion-weighted imaging (DWI) can be helpful in identifying premenopausal ovaries that show moderately high signal intensity. One point worth noting is that in postmenopausal women, ovaries may be misdiagnosed as a lymph node due to absence of follicles. The location of ovaries internally to internal iliac vessels may help for the differentiation with obturator lymph node that are located externally. On the other hand, the absence of a normal ipsilateral adnexa around a lesion suggests that adnexal origin is likely [8, 9]. If normal ipsilateral adnexa are not found, it is important to evaluate the location and course of gonadal vessels [10, 11]. Typically, gonadal vessels are located anterior to the psoas muscles. If these vessels lead to the pelvic lesion, it is likely of adnexal origin.
2. When the lesion abuts normal adnexa, various imaging signs described previously can help assess the relationship between the lesion and adjacent organs [10] (Fig. 3).

**Table:** 

The “beak sign” is defined as sharp angles between the adnexa and the lesion, causing the edges of the ovary to deform into a beak shape. The presence of the beak sign suggests that the lesion originates from the ovary [10, 12]. The “bridging vessel” sign and “claw sign” are indicative of the lesion originating from the uterus. The bridging vessel sign refers to vessels extending between the uterus and the lesion, as observed in pedunculated uterine leiomyomas [13]. The “claw sign” corresponds to the uterine tissue draping over the lesion and is also typically observed in uterine leiomyomas [14].	

### Pitfall 2

An O-RADS MRI score of 1 includes physiological observations which must not be mistaken for a lesion. These physiologic observations include (1) Follicle, defined as a unilocular simple cyst ≤ 3 cm in premenopausal women and a (2) corpus luteum, a corpus luteum arises at the site of a follicle after ovum release and often shows a crenulated wall due to infoldings. Corpus luteum is characterized as a cyst ≤ 3 cm with a thick, enhancing wall and can be simple or contain hemorrhage [15].

**Fig. 4:**
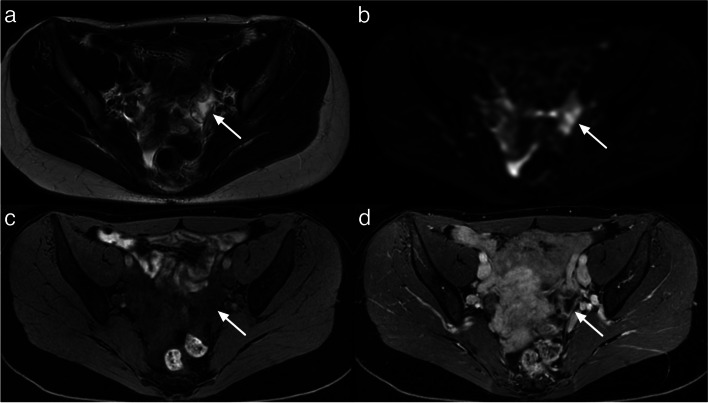
Example of MRI appearance of a corpus luteum cyst. **a** Axial T2-weighted sequence. **b** Axial DWI abdominal and pelvic scan (*b*: 1200 s/mm^2^). **c** Axial unenhanced T1-weighted water sequence. **d** Axial late-gadolinium T1-weighted sequence. Left ovarian corpus luteum cyst (maximal diameter: 35 mm) (white arrow)

Typically, corpus luteum cysts appear as hypointense on T1-weighted images (T1WI) and heteregeneously hyperintense on T2-weighted images (T2WI) with a thick, often crenulated wall showing high signal on diffusion-weighted imaging (DWI) and avid contrast enhancement [16, 17] (Fig. 4).

There can be variations in the appearance of corpus luteum, such as T1 hyperintensity and variable signal intensity on T2WI due to internal blood products. Uncommon findings may include multifocality, bilaterality, and rupture. Rupture can lead to substantial hemoperitoneum, visible as heterogeneous or T1-weighted hyperintense ascites, along with a focal interruption of the cyst wall [16, 17].

Differential diagnosis of corpus luteum includes:

- Endometrioma: Endometriomas are typically larger and demonstrate homogeneous T1-weighted hyperintensity and variable T2-weighted hypointensity (known as “T2 shading”), with a thin enhancing wall. Endometriomas result from chronic repetitive bleeding over multiple menstrual cycles, which is not observed with a corpus luteum [18–20].
- Tubo-ovarian abscess: Tubo-ovarian abscesses are typically bilateral and asymmetrical (due to different degree of evolution of pelvic inflammatory disease). They usually demonstrate thick enhancing walls, tubular cystic components, and surrounding fatty infiltration/stranding. Pus induces a very high signal intensity on DWI and a low signal intensity on ADC map. Differentiation of tubo-ovarian abscess from corpus luteum is also based on clinical presentation. In addition to pelvic pain, patients with tubo-ovarian abscesses often have fever and elevated white cell count [21, 22].

### Pitfall 3

**Fig. 5:**
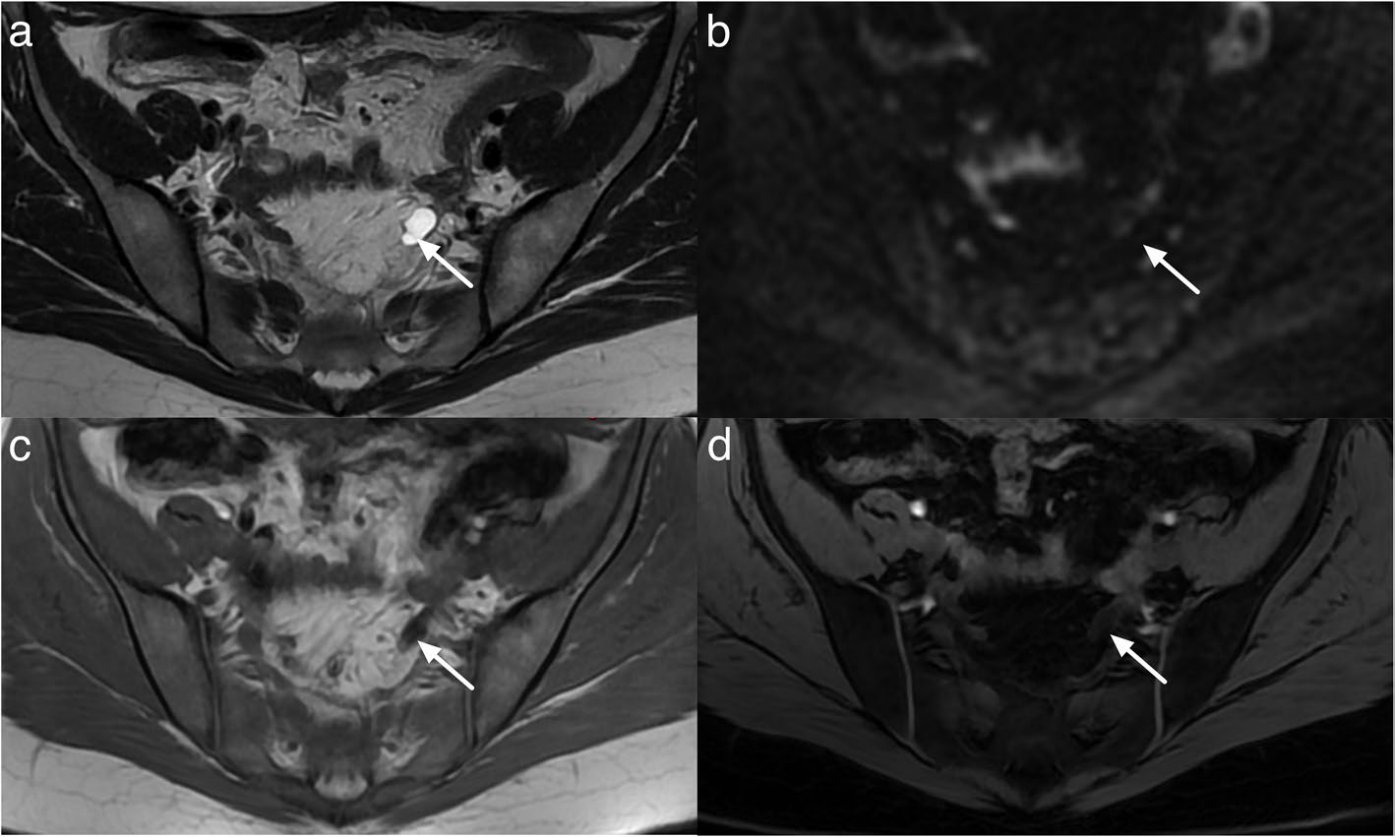
Example of MRI appearance of a para-ovarian cyst. **a** Axial T2-weighted sequence. **b** Axial DWI lombo-pelvic scan (*b*: 1200 s/mm^2^). **c** Axial unenhanced T1-weighted in-phase sequence. **d** Axial late-gadolinium T1-weighted sequence. Left para-ovarian cyst (maximal diameter: 17 mm) white arrow

Of note, a para-ovarian cyst is considered O-RADS MRI score 2 and not 1 as it is not a physiological lesion. Para-ovarian cysts originate from the mesosalpinx. Para-ovarian cysts are non-cancerous cysts that originate in the broad ligament and constitute approximately 10–20% of all adnexal lesions. When observed on MRI, these cysts typically appear as simple unilocular structures with low signal intensity (SI) on T1-weighted images (T1WI) and high signal intensity on T2-weighted images (T2WI). They exhibit a thin outer wall, measuring less than 3 mm, and are located adjacent to the ovary but remain separate from it [23] (Fig. 5).

*O-RADS MRI score 2*: This category includes adnexal lesions that are almost certainly benign with a PPV for malignancy of less than 0.5%. It should be noted that observations scored as category 2 or higher represent non-physiologic lesions. The term "lesion" is preferred to "masses", "tissues" and  "tumor" and should be adopted for standardization [1].

**Table:** 

**Definition:** An adnexal *lesion* is defined as an adnexal observation which does not meet criteria for a physiologic finding. This is subdivided further into cystic lesion without solid tissue, cystic lesion with solid tissue, and solid lesion. Cystic lesions can be unilocular or multilocular. Purely solid lesions are defined as having less than 20% cystic component. *O-RADS MRI score 2* includes: -Unilocular cyst with no wall enhancement and no enhancing solid tissue If no wall enhancement, may have any type of fluid content. If smooth wall enhancement, may have simple or endometriotic fluid content. -Lesion with lipid content and: No enhancing solid tissue. Only a small amount of enhancing tissue (Rokitansky nodule). -Lesion with homogenously hypointense solid tissue on T2WI and DWI. -Dilated fallopian tube with simple fluid content (hydrosalpinx) and no enhancing solid tissue. May demonstrate a thin, smooth wall/endosalpingeal folds with enhancement. -Para-ovarian cyst with no enhancing solid tissue. May contain any type of fluid content, and may have a thin, smooth wall +/− enhancement.	

### Pitfall 1

It is important in this category to recognize *simple fluid versus non-simple fluid* within a cyst. Non-simple fluid may be endometriotic, hemorrhagic, proteinaceous, or lipid in composition [24].

### Pearls 1

*Simple fluid* demonstrates the same signal intensity as cerebrospinal fluid (CSF) on all sequences.

**Fig. 6:**
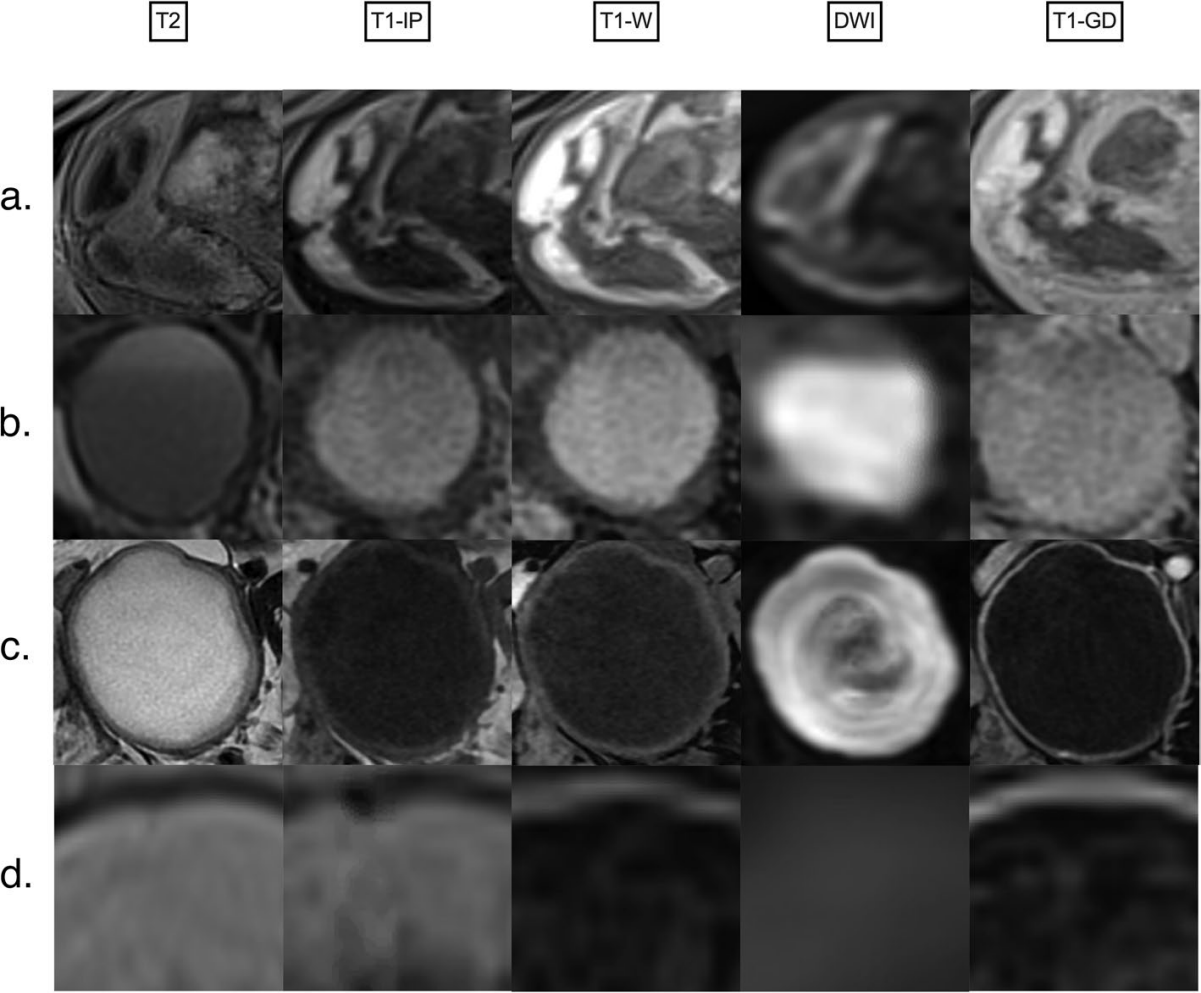
MRI assessment of non-simple fluid. First column corresponding to T2-weighted signal. Second column corresponding to T1-weighted in-phase sequence. Third column corresponding to T1-weighted water-phase sequence. Fourth column corresponding to diffusion-weighted sequence. Last column corresponding to late gadolinium T1-weighted sequence. **a** Hemorrhagic fluid. **b** Endometriotic fluid. **c** Proteinaceous fluid in this case: pus inside an abscess. **d** Lipid-containing fluid

*Non-simple fluid* can be characterized using the following lexicon descriptors:

- *Hemorrhagic fluid*: The signal intensity can be heterogeneous and variable depending on the age of blood products. For instance, late subacute hemorrhage appears hyperintense on T1-weighted images (T1WI) and T2-weighted images (T2WI) (Fig. 6).
- *Endometriotic fluid*: Typically appears homogeneously hyperintense on T1WI, with corresponding hypointensity on T2WI, commonly known as “T2 shading.” Only endometriotic fluid may have higher T1W signal than fatty content. Ancillary findings of endometrioma include multiplicity and the presence of “T2 dark spots,” representing blood clots, which support the diagnosis of endometrioma and aid in distinguishing it from hemorrhagic fluid (Fig. 6).
- *Proteinaceous fluid*: Fluid composed of mucin, colloid, or purulent material may display variable high T1W signal (Fig. 6).
- *Fat- or lipid-containing fluid*: Expected to appear hyperintense on both T1W and T2W images, with signal loss on fat-saturated sequences. Notably, the presence of intravoxel fat as detected on chemical shift T1WI out-of-phase sequences, even without detectable macroscopic fat, still represents “lipid-containing fluid” (i.e., dermoid) (Fig. 6).

To recognize non-simple fluid, it is essential to always check T1WI without and with fat saturation, T1 in- and opposed phase, T2WI, and DWI.

### Pitfall 2

**Fig. 7:**
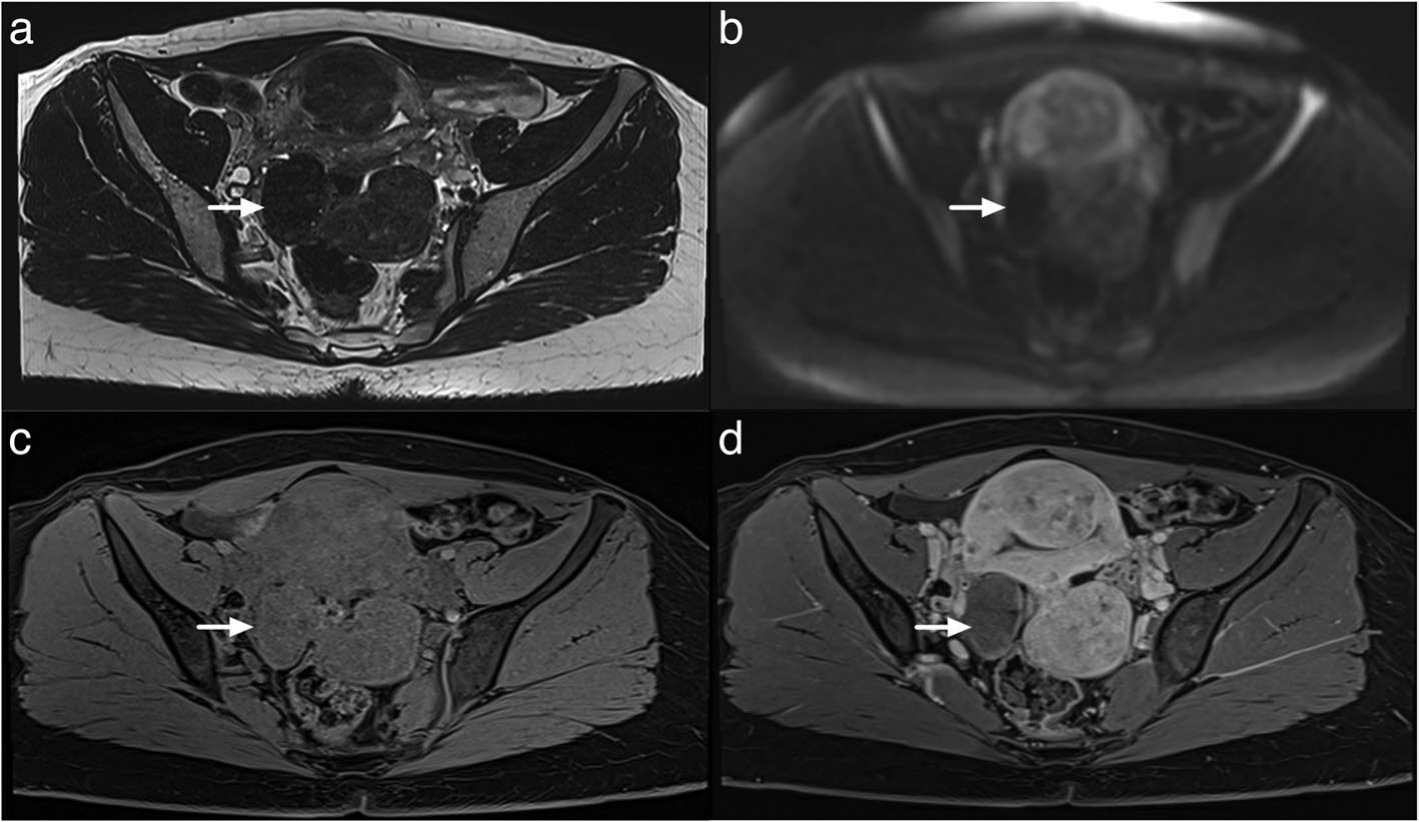
Illustration of the dark T2/DWI appearance on MRI. **a** Axial T2-weighted sequence. **b** Axial DWI abdominal and pelvicscan (*b*: 1200 s/mm^2^). **c** Axial unenhanced T1-weighted in-phase sequence. **d** Axial late gadolinium T1-weighted sequence. Right ovarian lesion classified O-RADS2 corresponding to a right ovarian fibroma (maximal diameter: 47 mm) (white arrow)

Identification and proper classification of “dark T2/dark DWI” lesions is crucial in MRI evaluation (Fig. 7) [25–27]. The ACR O-RADS MRI committee introduced the term “dark T2/dark DWI” to describe lesions composed of fibrous tissues showing uniformly hypointense signal intensity on both T2-weighted and high-*b*-value DWI scans. Note, this only refers to the solid component in the lesion.

Adnexal lesions containing solid tissue and displaying characteristic homogenous dark T2/dark DWI signal intensity on MRI are categorized as O-RADS MRI 2 score, regardless of their enhancement characteristics. These lesions are benign, commonly representing either fibroma, fibrothecoma, Brenner tumor, or cystadenofibroma [27] (Fig. 7).

Pearls to correctly interpret findings on DWI [25, 26]:

✓ Using an optimal high b-value of at least 1000 s/mm^2^.
✓ Defining dark DWI as having the same signal intensity as simple fluid on high-b-value images (e.g., urine in the bladder), where premenopausal ovaries are moderately bright.
✓ Comparing the DWI signal intensity of the lesion with that on T2WI and ADC images.
✓ Applying the “dark T2/dark DWI” categorization for O-RADS 2 only if the entire lesion appears homogeneously dark on T2 and high-b-value DWI. If the lesion shows foci of restricted diffusion or intermediate T2 signal intensity within an otherwise dark T2/dark DWI lesion, it should not be categorized as O-RADS MRI Score 2.
✓ Restricted diffusion is defined as high signal intensity on high-b-value DWI with low signal intensity on the ADC map. However, this characteristic does not offer effective risk stratification for adnexal lesions as there is substantial overlap between benign and malignant lesions. The role of ADC quantification to refine O-RADS MRI score 4 remains an area of active research.

### Pitfall 3

**Fig. 8:**
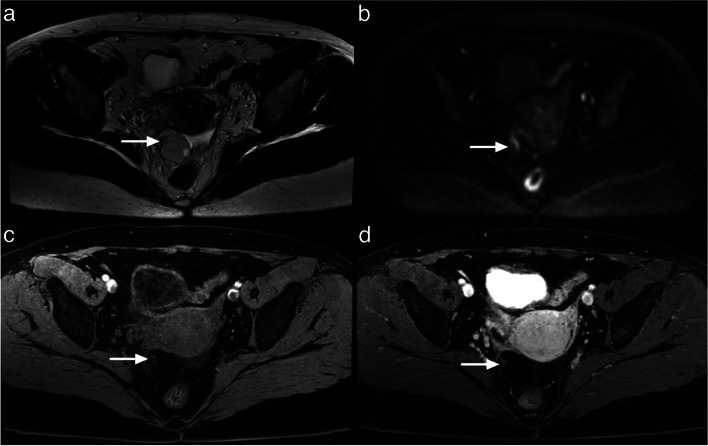
Example of MRI appearance of a Rokitansky nodule which does not account for a solid tissue. **a** Axial T2-weighted sequence. **b** Axial DWI abdominal and pelvic scan (*b*: 1200 s/mm^2^). **c** Axial unenhanced T1-weighted in-phase sequence. **d** Axial late-gadolinium T1-weighted sequence. Right ovarian dermoïd cyst with a small Rokitansky nodule (white arrow)

Lesions with lipid content and no or small amount of solid tissue (Rokitansky nodule) are classified as O-RADS MRI score 2 [28] (Fig. 8).

*O-RADS MRI score 3*: This category carries a low-risk for malignancy with a PPV of about 5%.

 O-RADS MRI score 3 lesions include

**Table:** 

**Definition of O-RADS MRI score 3:** -Unilocular cyst with proteinaceous, hemorrhagic, or mucinous fluid content and No enhancing solid tissue Smooth wall enhancement -Multilocular cyst with any type fluid content No enhancing solid tissue No lipid content May have smooth enhancing septae and wall -Lesion with solid tissue (excluding dark T2/dark DWI) with low-risk time intensity curve (TIC) on DCE MRI. -Dilated fallopian tube with no enhancing soft tissue nodule and: Non-simple fluid and thin walls/folds Simple fluid with thick, smooth walls/folds	

Significant efforts have been undertaken to clarify the lexicon terms “solid tissue” and “solid component,” which were previously used interchangeably and led to errors in the EURAD study, accounting for 63 out of 139 misclassified adnexal lesions [4]. The term “solid component” refers to any non-fluid component of a lesion and can be categorized into two types:

1. Solid components without enhancement, such as clot, hairs, calcifications, or debris.
2. Solid components with enhancement, including enhanced solid components that do not correspond to solid tissue, such as smooth thin or thick wall or septations, Rokitansky nodules, and endosalpingeal folds.

On the other hand, “solid tissue” is defined as exhibiting contrast enhancement and conforming to specific morphologies, namely papillary projections, mural nodules, irregular septations/walls, and larger solid portions.

A study by Thomassin-Naggara et al. found that 67 benign pelvic lesions were falsely classified as O-RADS MR 4 (false positive). Among these cases, over half (50.7%, 34/67) resulted from the misunderstanding of how solid tissue is defined in the lexicon [7].

**Fig. 9:**
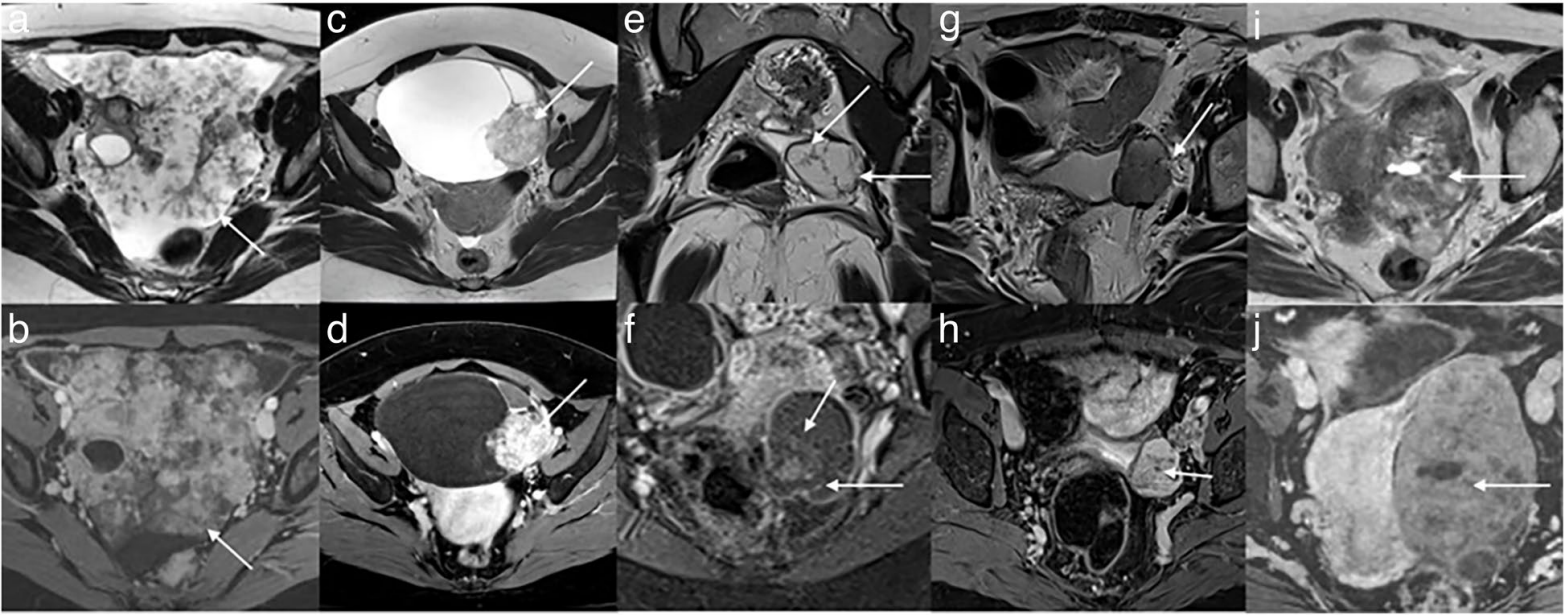
Example of solid tissue descriptors: **a** Axial T2-weighted sequence without fat saturation. **b** Axial unenhanced T1-weighted Water sequence showing papillary projections in bilateral serous borderline ovarian lesions (arrows). **c** Axial T2-weighted sequence without fat saturation. **d** Axial unenhanced T1-weighted Water sequence showing a large mural nodule (arrows) in a high-grade serous ovarian cancer. **e** Axial T2-weighted sequence without fat saturation. **f** Axial unenhanced T1-weighted Water sequence showing irregular septations (arrows) in bilateral serous borderline ovarian lesions. **g** Axial T2-weighted sequence without fat saturation. **h** Axial unenhanced T1-weighted Water sequence showing irregular wall (arrows) in a serous borderline bilateral ovarian tumor. **i** Axial T2-weighted sequence without fat saturation. **j** Axial unenhanced T1-weighted Water sequence showing irregular septations showing large solid component (arrows) high-grade serous ovarian cancer

Pearls: What is included in the definition of a solid tissue? (Fig. 9)

**Table:** 

• *Papillary projection*: An enhancing solid component emerging from the inner or outer wall or septation, displaying a branching architecture. • *Mural nodule*: An enhancing solid component measuring greater than 3 mm, originating from the wall or septation, with a nodular appearance. • *Irregular septation*: An enhancing linear strand extending from one internal surface of the cyst to the contralateral side, demonstrating an uneven margin. • *Irregular wall*: An enhancing cyst wall displaying an uneven margin. • *Larger solid portion*: An enhancing component of an adnexal lesion that does not fit into the categories of papillary projection, mural nodule, or irregular septation/wall.	

### Pitfalls 1: What is not considered solid tissue?

**Fig. 10:**
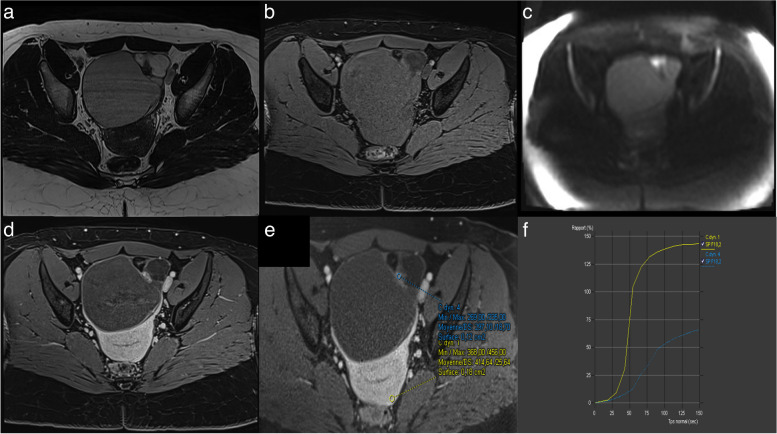
Example of mature cystic teratoma. **a** Axial T2-weighted sequence without fat saturation. **b** Axial unenhanced T1-weighted Water sequence. **c** Axial DWI scan (*b*: 1200 s/mm^2^). **d** Axial late-gadolinium T1-weighted sequence. **e**, **f** DCE T1-weighted sequence with corresponding curve of the external myometrium: yellow curve and Rokitansky protuberance: purple curve corresponding to a type I curve
- Endosalpingeal folds (Fig. 11). Another common cause of false positives is the misinterpretation of endosalpingeal folds as papillary projections, especially in the context of pelvic inflammatory disease (PID). PID is typically associated with inflammatory processes, leading to thickening, and marked enhancement of the fallopian tube wall and endosalpingeal folds. A study by Thomassin-Naggara et al. reported that 11 out of 12 PID cases were incorrectly scored as O-RADS MRI category 4 or 5 because the TIC analysis was performed by placing a region of interest (ROI) on endosalpingeal folds or thickened wall [7]. Radiologists should exercise caution and consider all imaging findings, such as premenopausal status, tubular shape, inflammatory changes in adjacent structures, and the presence of pus within the lesion. Pus can be recognized as high signal intensity on high-b-value DWI with a corresponding low signal intensity on the ADC map.

- Fat, clot, debris. Use of dedicated axial unenhanced T1-weighted in-, opposed-phased, fat and water sequence and subtraction images are particularly helpful in this setting.
- Normal ovarian parenchyma
- Rokitansky nodule (Figs. 8 and 10). Rokitansky nodule is a small solid component that may show enhancement, but it is not categorized as solid tissue. Thomassin-Naggara et al. reported that twenty mature cystic teratomas were misclassified, with 19/20 classified as O-RADS 4 or 5. These errors occurred because readers examined TIC and recorded intermediate or high-risk TIC in a Rokitansky nodule which should not considered soft tissue [7]. Rokitansky nodule can demonstrate enhancement due to the presence of smooth muscular cells and fibrous, neuroglial, or thyroid tissue [28].

**Fig. 11:**
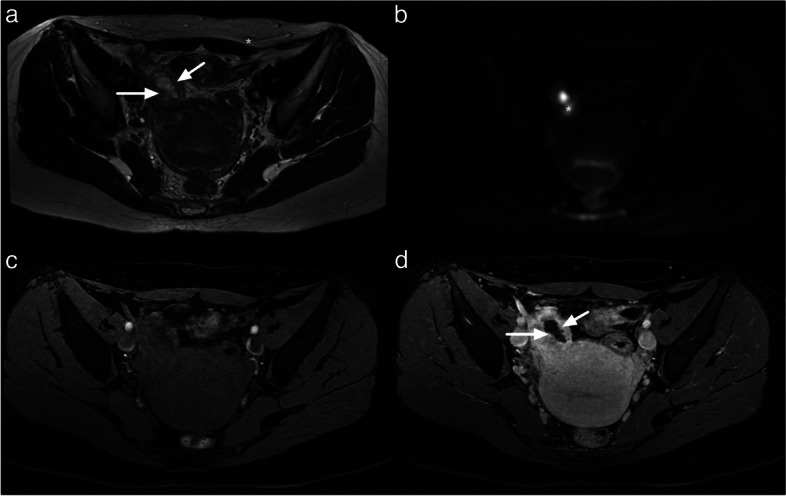
Example of MRI appearance endosalpingial folds. **a** Axial T2-weighted sequence. **b** Axial DWI lombo-pelvic scan (*b*: 1200 s/mm^2^). **c** Axial unenhanced T1-weighted water sequence. **d** Axial late-gadolinium T1-weighted sequence. Multiple endosalpingial folds (white arrow) potentially leading to a misinterpretation with ovarian vegetations. Note the hypersignal on the DWI scan corresponding to pus inside the fallopian tube (white star)
- Fimbriated end of the tube may look like irregular solid tissue with strong enhancement and may be recognized thanks to its stellar morphology (Fig. 12)

**Fig. 12:**
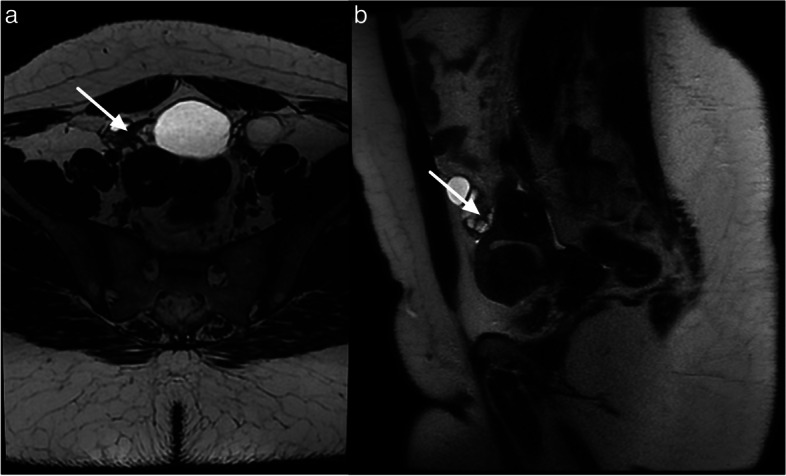
Fimbrial end of the tube between a dilated hydrosalpynx and the right ovarian structure. **a** Axial T2-weighted sequence. **b** Sagittal T2-weighted sequence. Stellar morphology of the fimbrial end of the tube (white arrow)
- Smooth wall or septa (Fig. 13) as opposed to irregular wall or septa.

**Fig. 13:**
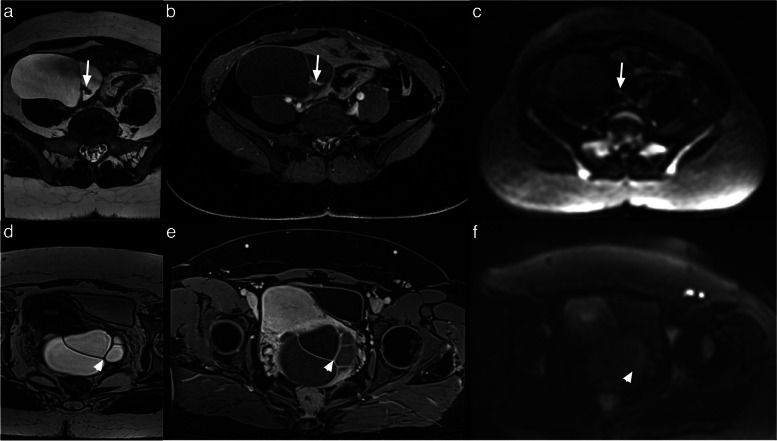
MRI appearance of a thick (first row, white arrow) and a thin septa (second row). **a** Axial T2-weighted sequence showing a thickened septa with tissular component. **b** Axial late-gadolinium T1-weighted sequence showing a tissular enhancement. **c** Axial DWI abdominal and pelvic scan (*b*: 1200 s/mm^2^) with hypersignal of the tissular component. **d** Axial T2-weighted sequence showing a thin septa. **e** Axial late-gadolinium T1-weighted sequence showing a thin enhancement. **f** Axial DWI abdominal and pelvic scan (*b*: 1200 s/mm^2^) showing no hypersignal

### Pitfalls 2: Failure to recognize a low-risk TIC curve

**Fig. 14:**
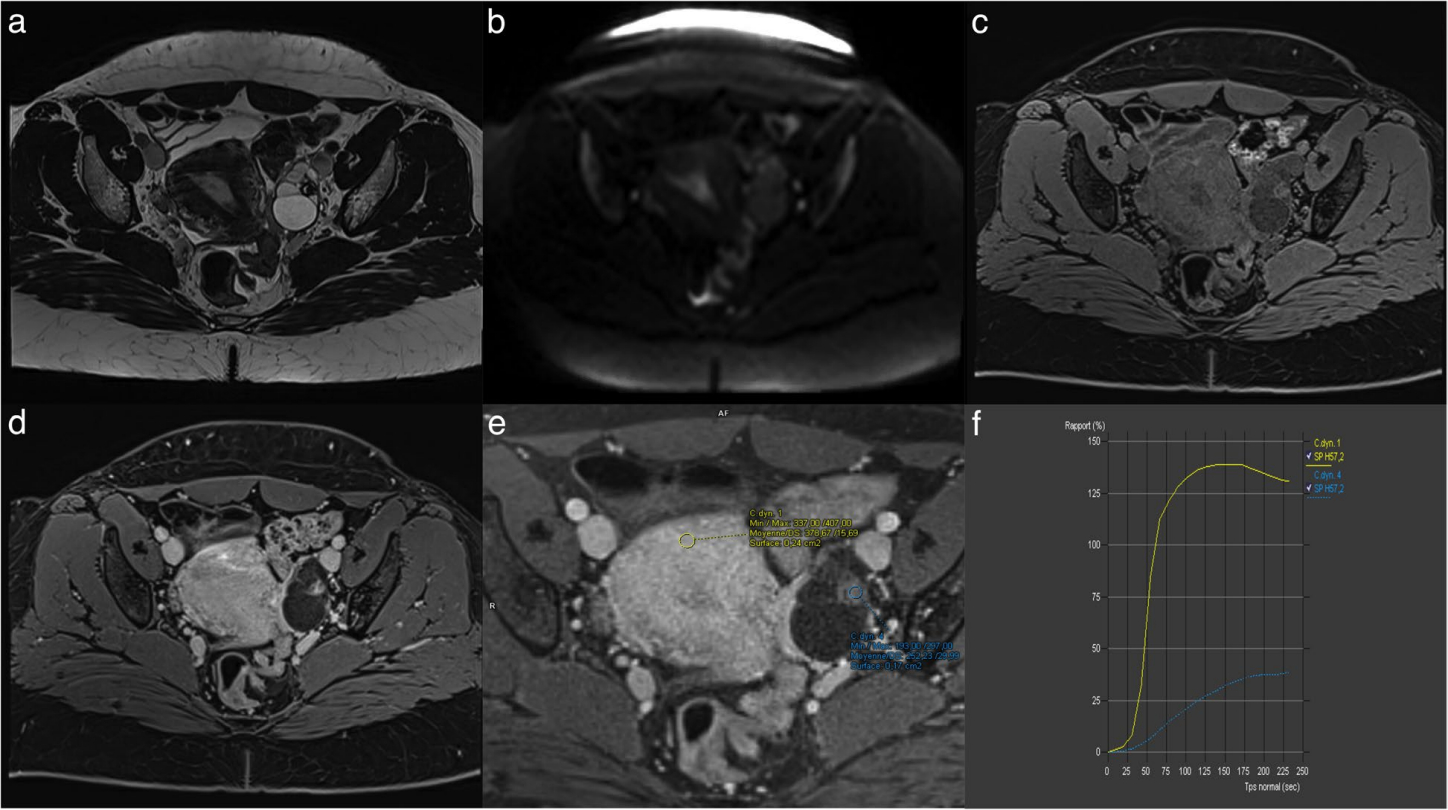
Example of left ovarian lesion with a solid component with a type I dynamic enhancement curve. **a** Axial T2-weighted sequence without fat saturation. **b** Axial DWI scan (*b*: 1200 s/mm^2^). **c** Axial unenhanced T1-weighted water sequence. **d** Axial late-gadolinium T1-weighted sequence. **e**, **f** DCE MRI T1-weighted sequence with corresponding curve of the tissular component showing a type I curve. This was favored to be a benign cystadenofibroma

**Fig. 15:**
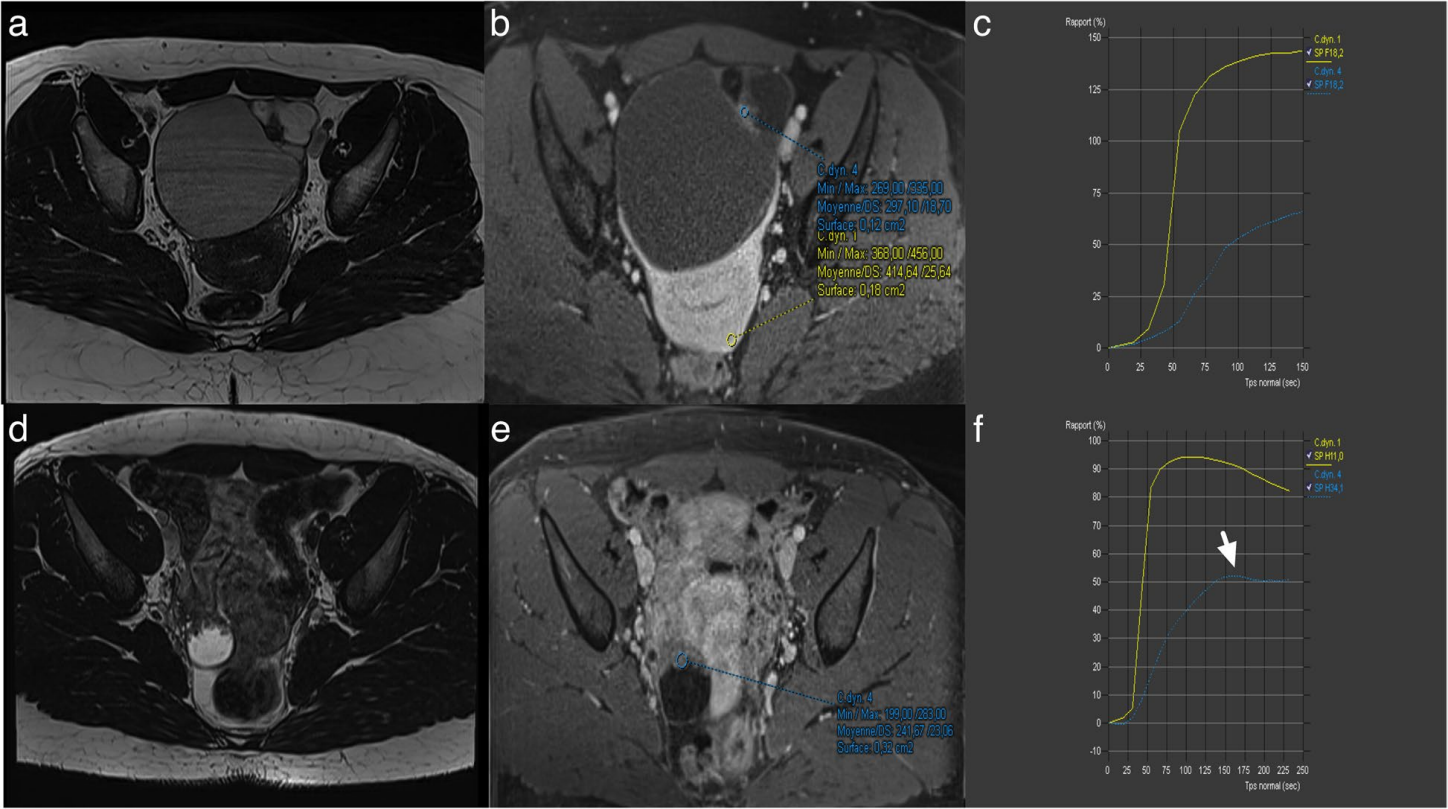
Difference between a type I curve and a type II curve. Same case as illustrated previously: **a** Axial T2-weighted sequence without fat saturation. **b,c** DCE MRI T1-weighted sequence with corresponding curve of the tissular component showing a type I curve corresponding to a cystadenofibroma. Other case with (**d**) Axial T2-weighted sequence without fat saturation. **e**, **f** DCE MRI T1-weighted sequence with corresponding curve of the tissular component showing a type II curve corresponding to a borderline serous cystadenoma

A low-risk TIC curve is characterized by slow and gradual enhancement over time with no well-defined shoulder or plateau (Fig. 14). Some common errors involve difficulty in distinguishing the shoulder and plateau between low- and intermediate-risk TICs, while no confusion was observed between high-risk TICs versus intermediate and low-risk TICs. This misinterpretation led to 7 false positives and 4 false negatives, all of which were correctly identified by the blind readers who strictly adhered to the score rules [4]. As per the lexicon, a low-risk TIC (type 1 curve) does not exhibit a shoulder or plateau, even if the slope is acute, whereas an intermediate-risk TIC (type 2 curve) demonstrates a plateau and an initial slope that is less steep than that of the outer myometrium (Fig. 15) [4, 7, 29, 30].

#### O-RADS MRI score 4 and 5

**Table:** 

**Definition of O-RADS MRI score 4:** Adnexal lesions with an intermediate risk for malignancy as defined by a PPV of approximately 50%. **O-RADS MRI score 4 lesions include:** -Lesion with solid enhancing tissue (except for dark T2/dark DWI) with: Intermediate-risk TIC on DCE MRI. (If DCE MRI is not feasible, any lesion with enhancement ≤ myometrium at 30–40 s on non-DCE MRI. This analysis is less accurate than TIC analysis with a loss of specificity as no difference is feasible in the presence or absence of a plateau). -Lesion with lipid content and large volume enhancing solid tissue.	

**Table:** 

**Definition of O-RADS MRI score 5:** Lesions in this category are considered high-risk with a PPV for malignancy of about 90%. **O-RADS MRI score 5 lesions include:** -Lesion with solid tissue (except for dark T2/dark DWI) with: High-risk TIC on DCE MRI. (If DCE MRI is not feasible, any lesion with enhancement > myometrium at 30–40 s on non- DCE MRI. This analysis has the same accuracy as TIC analysis). -Peritoneal, mesenteric, or omental nodularity or irregular thickening with or without ascites.	

### Pearls

**Fig. 16:**
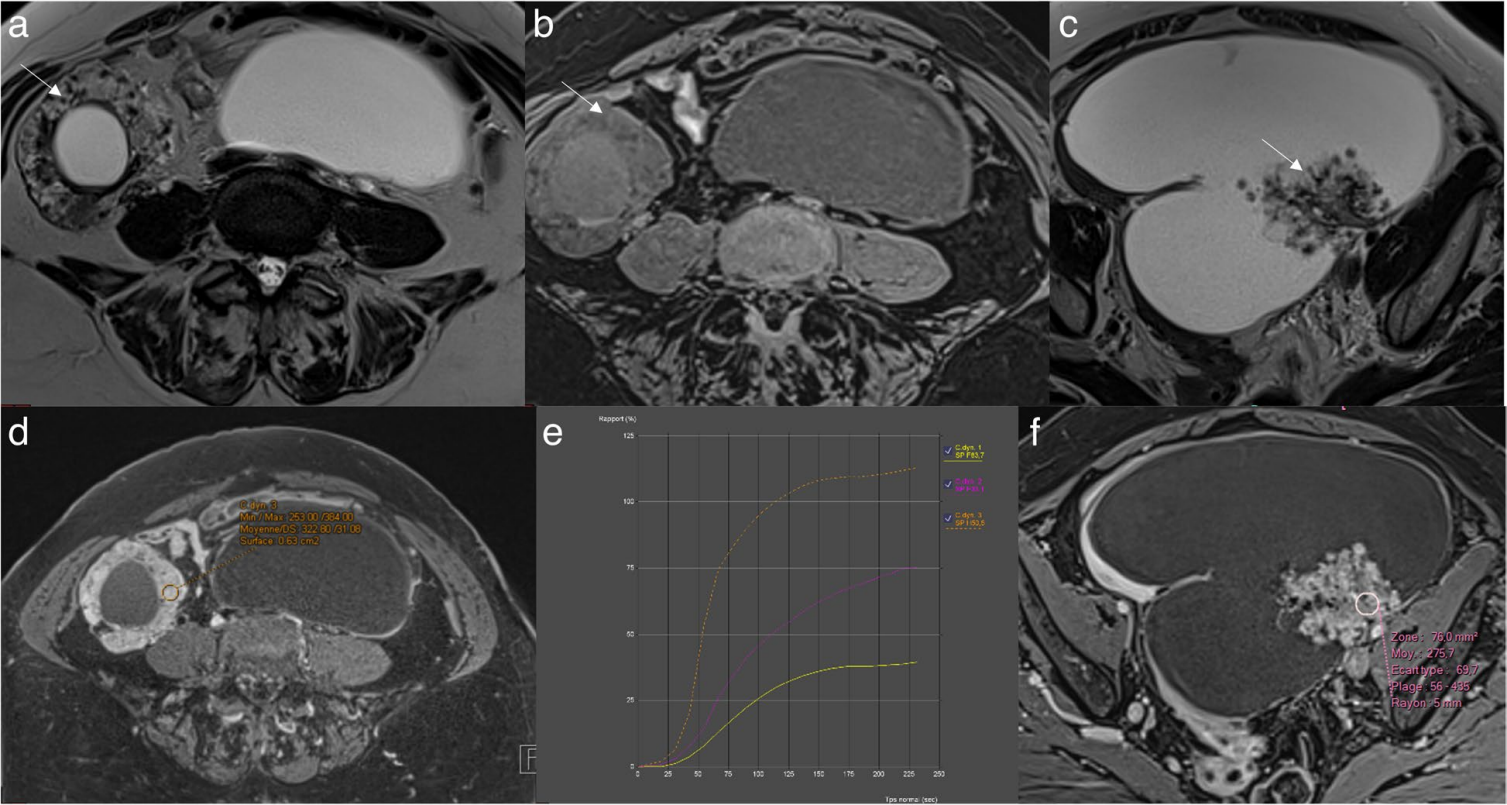
Example of bilateral ovarian lesion with a solid tissue with a type II dynamic enhancement curve (**e**) and type III dynamic enhancement curve (**e**). **a** Axial T2-weighted sequence without fat saturation and (**b**) Axial unenhanced T1-weighted water sequence show a mixed right cystic and solid lesion with (**c**) large enhancing solid tissue portion (**d**) with corresponding curve of the tissular component showing a type III curve (orange curve) (**e**) consistent with an O-RADS MRI 5 score. **c** Axial T2-weighted sequence without fat saturation and (**f**) DCE MRI T1-weighted sequence show a large left cystic lesion with solid tissue represented by papillary projections (arrow), corresponding to a type II curve (pink curve). Note the myometrium is represented by the yellow curve. The hypothesis of a low-grade serous ovarian cancer was made and later proven on histology

The use of DCE technique is recommended for the optimal evaluation of enhancement characteristics in adnexal lesions [4, 7, 29, 30]. This involves employing a 3-D T1WI fat-saturated sequence with a spatial resolution of 3 mm and temporal resolution of 15 s. During DCE acquisition, a region of interest is placed on the earliest enhancing region of the solid tissue to generate a Time-Intensity Curve (TIC). A second region of interest is placed on the outer myometrium, serving as a reference standard, while avoiding the arcuate vessels. The analysis is performed in percentage of enhancement or relative enhancement, requiring the initial phases of DCE MR to be obtained before the start of intravenous contrast injection. Three types of TICs have been defined [4, 7, 29, 30]. A low-risk or Type 1 curve demonstrates a gradual increase in enhancement over time without a shoulder or plateau, previously defined in O-RADS MRI 3 score (Fig. 14) [4, 7, 29, 30]. An intermediate risk or type 2 curve demonstrates moderate enhancement within the adnexal lesion, with an initial slope, followed by a shoulder and a plateau that is less than or equal to the myometrium (Fig. 15) [4, 7, 29, 30]. On the other hand, a high-risk or Type 3 curve exhibits a steeper initial slope than the myometrium, followed by a shoulder and a plateau, and the maximal enhancement may be higher or lower than the external myometrium (Fig. 16) [4, 7, 29, 30].

In cases where DCE is not available, a non-dynamic contrast-enhanced MRI may be obtained, including pre-contrast images and images acquired at 30 to 40 s after intravenous contrast injection. This helps in evaluating the relative enhancement of the solid component with the adnexal lesion compared to the outer myometrium, aiding in lesion classification (O-RADS MRI score 3 versus O-RADS MRI scores 4 and 5) [1, 24].

If the uterus is absent, differentiation between intermediate- and high-risk TICs becomes challenging, but a low-risk TIC can still be identified as showing progressive enhancement without a plateau [1, 24].

Specific tips to avoid curve pitfalls:

- When dealing with small amounts of solid tissue, be cautious about motion artifacts that may affect the region of interest (ROI) in each phase. Note, a small amount of solid tissue is defined as solid tissue measuring up to 3 mm.
- In cases of weak enhancement, consider evaluating the presence of a potential plateau before comparing it to the outer myometrium.
- For mixed solid tissue with cystic content demonstrating high signal intensity on T1WI, it is beneficial to examine subtraction images.

## Conclusion

The ACR Reporting and Data Systems (RADS) offer a standardized framework for characterizing and reporting imaging findings. The primary goal is to standardize terminology, minimize misinterpretation, and enhance communication between radiologists and referring clinicians. These goals can be accomplished though radiologist training and dissemination of knowledge about the lexicon and the use of risk scores. Similar to other RADS, proper application of O-RADS MRI system requires extensive training to facilitate its widespread use and accurate implementation in daily clinical practice.

## Data Availability

Not applicable.

## Abbreviations

ACR: American College of Radiology
DCE: Dynamic contrast enhanced
DWI: Diffusion-weighted imaging
IOTA: International Ovarian Tumor Analysis
MRI: Magnetic resonance imaging
O-RADS: Ovarian-Adnexal Reporting and Data Systems
TIC: Time-Intensity Curve
WI: Weighted imaging
